# Mixed Small-Molecule Matrices Improve Nanoparticle Dispersibility in Organic Semiconductor-Nanoparticle Films

**DOI:** 10.1021/acs.langmuir.3c00152

**Published:** 2023-03-20

**Authors:** Daniel T. W. Toolan, Michael P. Weir, Rachel C. Kilbride, John E. Anthony, Neil C. Greenham, Richard H. Friend, Akshay Rao, Oleksandr O. Mykhaylyk, Richard A. L. Jones, Anthony J. Ryan

**Affiliations:** † Department of Chemistry, Brook Hill, The University of Sheffield, Dainton Building, Sheffield S3 7HF, U.K.; ‡ Department of Physics and Astronomy, The University of Sheffield, Hicks Building, Hounsfield Road, Sheffield S3 7RH, U.K.; § School of Physics and Astronomy, The University of Nottingham, University Park, Nottingham NG7 2RD, U.K.; ∥ University of Kentucky Center for Applied Energy Research, 2582 Research Park Drive, Lexington, Kentucky 40511, United States; ⊥ Cavendish Laboratory, Cambridge University, J. J. Thomson Avenue, Cambridge CB3 0HE, U.K.; # John Owens Building, The University of Manchester, Oxford Road, Manchester M13 9PL, U.K.

## Abstract

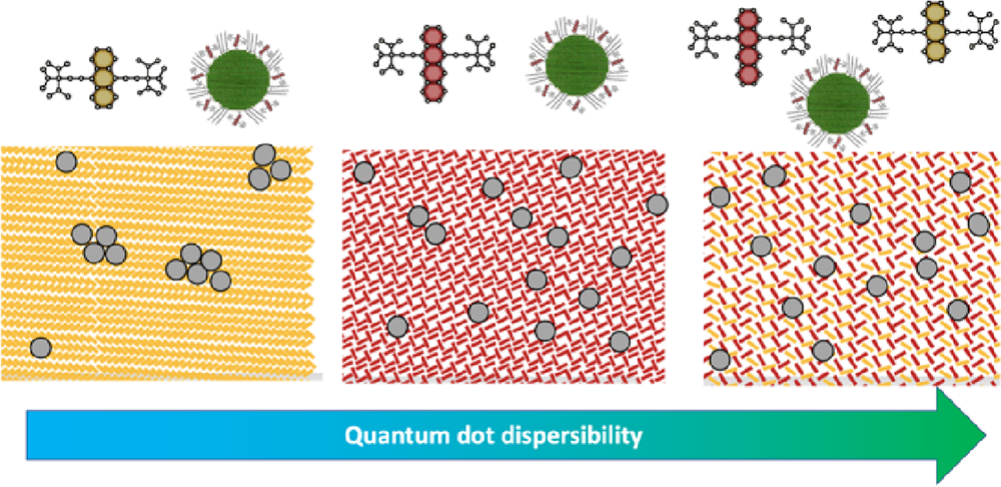

Controlling the dispersibility of nanocrystalline inorganic quantum dots (QDs) within organic semiconductor (OSC):QD nanocomposite films is critical for a wide range of optoelectronic devices. This work demonstrates how small changes to the OSC host molecule can have a dramatic detrimental effect on QD dispersibility within the host organic semiconductor matrix as quantified by grazing incidence X-ray scattering. It is commonplace to modify QD surface chemistry to enhance QD dispersibility within an OSC host. Here, an alternative route toward optimizing QD dispersibilities is demonstrated, which dramatically improves QD dispersibilities through blending two different OSCs to form a fully mixed OSC matrix phase.

## Introduction

Quantum dot (QD) nanocomposites, where QDs are dispersed within a host material, find applications in solar cells,^1−4^ light-emitting diodes,^5,6^ photodetectors, photon upconversion,^7^ and, recently, photon multipliers.^8^ In these applications, ligands bound to the QD surface not only determine QD dispersibility in the surrounding (host) material but also influence both the electronic and optical properties.

As-synthesized QDs are typically functionalized with ligands, which do not enable electronic coupling between the host and QD, such as oleate or trioctylphosphine oxide. This can be overcome through ligand exchange approaches utilizing short ligands (e.g., pyridine, butylamine, and hexanoic acid) or by removing the QD ligands via thermal annealing.^9−12^ A further approach has been the in situ synthesis of QDs without stabilizing organic ligands inside the host material.^13^ Such approaches often generate nanocomposites containing poorly dispersed, phase-segregated QDs.^13−15^

A successful surface chemistry approach for achieving good QD dispersibility within a host material has been through ligand exchange approaches utilizing ligands that are chemically similar to the host material. This has been demonstrated in QD:polymer,^16^ QD:perovskite,^17^ and QD:organic semiconductor (OSC) materials.^18,19^

QD:small-molecule OSC nanocomposites are of particular interest as photon multiplierfilms due to their potential to enhance photovoltaic device efficiencies through converting high-energy (blue) photons into multiple lower-energy (red) photons better energetically matched to the photovoltaic bandgap, minimizing thermalization losses and enabling the Shockley–Queisser limit to be surpassed.^20−22^ The conversion of high-energy photons to multiple lower-energy photons utilizes singlet fission, a multiple exciton generation mechanism occurring in some OSCs, where one photoexcited singlet exciton forms two triplet excitons.^23^ The generated triplet excitons are then transferred from the OSC to the QD leading to photon emission.

Photon multiplication in QD:OSC nanocomposites has been demonstrated in the prototypical system comprising 5,12-bis((triisopropylsilyl)ethynyl)tetracene (TIPS-Tc) as the singlet fission-capable OSC and [6,11-bis((triisopropylsilyl)ethynyl)tetracene-2-carboxylic acid (TET-CA)-ligated lead sulfide QDs (PbS-TET-CA).^19^ PbS-TET-CA is therefore a QD species modified with a ligand, which is itself an OSC capable of singlet fission. This approach has a dual benefit: (i) the efficiency of exciton transfer from the organic semiconductor to the QD is improved; and (ii), as a result of more favorable interactions between the QD and the OSC matrix, the QD dispersibility is improved when compared to TIPS-Tc:PbS-oleate QD blends.^18,19^

While this approach shows significant promise, the energies of emitted photons from the TIPS-Tc:PbS-TET-CA are not yet fully optimized with the absorption profile of silicon photovoltaics. This places an unfavorable limit on any potential efficiency gains. In order to achieve the potential of the photon multiplier concept, there is a need to explore alternative singlet fission-capable host OSCs in order to develop photon multiplier systems that are better energetically matched to silicon photovoltaics. Moreover, we need to establish universal design rules that enable the generation of optimal QD dispersions within any host small-molecule organic semiconductor.

This work first confirms that changing the OSC host molecule from TIPS-Tc to either 9,10-bis((triisopropylsilyl)ethynyl)anthracene (TIPS-Ac) or 5,10-bis((triisopropylsilyl)ethynyl)-2-fluoroanthra[2,3-*b*]thiophene (TIPS-FTA) has a dramatic, detrimental effect on QD dispersibility within the host organic semiconductor matrix. The important breakthrough, however, is to demonstrate a method by which good QD dispersibility can be attained even in the presence of a matrix that is not well-matched with the QD ligand. This is achieved through blending two closely related organic semiconductors (TIPS-Tc and TIPS-Ac) and exploring their rich phase behavior. Results show that when a TIPS-Tc:TIPS-Ac co-crystal is formed (i.e., possessing a different structure to either of the pure materials), good PbS-TET-CA QD dispersibility is achieved. These findings are critical for the development of effective photon multiplier devices where poorly dispersed QDs would be ineffective due to a combination of aggregation-induced quenching and inefficient triplet transfer from the bulk OSC host to the QD.

## Experimental Section

### Materials

5,12-Bis((triisopropylsilyl)ethynyl)tetracene (TIPS-Tc) and 6,11-bis((triisopropylsilyl)ethynyl)tetracene-2-carboxylic acid (TET-CA) were synthesized as described previously.^24,25^ Bis((triisopropylsilyl)ethynyl)anthracene (TIPS-Ac) was purchased from Tokyo Chemical Industry. Full details of the synthesis of 5,10-bis((triisopropylsilyl)ethynyl)-2-fluoroanthra[2,3-*b*]thiophene (TIPS-FTA) are provided in the Supporting Information. All other chemicals were purchased from Sigma Aldrich and used as delivered.

### Quantum Dot Synthesis

The synthesis of PbS QDs with oleate ligands (OA) and subsequent ligand exchange with TET-CA were carried out using previously reported methods.^26,27^ Briefly, lead oxide (0.45 g), oleic acid (7 g), and 1-octadecene (10 g) were loaded in a three-necked flask and degassed at 110 °C for 2 h. Subsequently, the reaction flask was flushed with nitrogen and the temperature was lowered to 95 °C. A solution containing bis(trimethylsilyl)sulfide (210 μL) in 1-octadecene (5 mL) was rapidly injected into the lead precursor solution. The reaction flask was then allowed to cool down naturally to ambient temperature (∼25 °C). The QDs were first extracted by adding hexane and acetone followed by centrifugation. The QDs were further purified with hexane and acetone and then redispersed in toluene at a concentration of ∼100 mg mL^–1^. The resultant procedure produces QDs with bound oleate ligands and a proportion of physisorbed oleic acid ligands. The dispersion was filtered with a 0.45 μm PTFE syringe filter before ligand exchange. Ligand exchange procedures were performed in a nitrogen-filled glovebox. The oleate QDs were first diluted to a concentration of 20 mg mL^–1^. The TET-CA ligands (100 mg mL^–1^ dissolved in tetrahydrofuran, THF) were then added into the QD dispersion, with a QD:ligand mass ratio of 1:0.1. As TET-CA has poor solubility in toluene, extra THF was added to the mixture to prevent precipitation of the ligands (toluene:THF = 4:1). The mixture was stirred for 30 min. The exchanged QDs were then purified with toluene/acetone by centrifugation. Finally, the QDs were redispersed in toluene.

### Solution Scattering

Small-angle X-ray scattering measurements on QD solutions were carried out using a Xeuss 2.0 instrument equipped with an Excillum MetalJet liquid gallium X-ray source. Samples were measured in 2 mm external diameter borosilicate glass capillaries with a 0.01 mm wall thickness, with scattering data collected for 900 s using collimating slits of 0.5 × 0.6 mm (“high flux” mode). Scattering patterns were recorded on a vertically offset Pilatus 1M detector with a sample-to-detector distance of 559 mm as calibrated using a silver behenate standard to achieve a *q*-range of 0.025–1.0 Å^–1^. Data reduction was performed using the instrument-specific Foxtrot software, and before fitting was performed using *SasView*.^28^ Full details on the sphere–hard sphere scattering model employed to fit the experimental data are available in the Supporting Information.

### Film Preparation

Stock solutions of TIPS-Tc, TIPS-Ac, and TIPS-FTA were prepared in toluene (100 mg mL^–1^). Solutions were heated to 50 °C for 1 h and vortex-mixed prior to use. OSC:PbS-TET-CA blends were mixed by volume to prepare solutions containing a total OSC content of 100 mg mL^–1^. For the TIPS-Tc:TIPS-Ac:PbS-TET-CA blends (later referred to as Tc:Ac:QD series and by the shorthand Ac_*x*_ where *x* represents the weight fraction of TIPS-Ac), TIPS-Ac weight fractions of 0.17, 0.33, 0.5, 0.66, and 0.83 were prepared with PbS-TET-CA contents of 10 mg mL^–1^. Silicon substrates were cleaned with Decon and ethanol followed by three deionized water rinses. Fifty microliters of casting solutions was deposited on silicon substrates and spun-cast at 1500 rpm for 2 min. All samples were prepared in a nitrogen glovebox and stored under nitrogen for 24 h prior to X-ray scattering measurements.

### Grazing Incidence Small/Wide Angle X-ray Scattering

Grazing incidence small- and wide-angle X-ray scattering (GISAXS/GIWAXS) were performed on the Xeuss 2.0 instrument equipped with an Excillum MetalJet liquid gallium X-ray source. Alignment was performed on silicon substrates via three iterative height (*z*) and rocking curve (Ω) scans, with the final grazing incidence angle set to Ω = 0.2°. Scattering patterns were recorded on a vertically offset Pilatus 1 M detector with a sample-to-detector distance of 332 mm, calibrated using a silver behenate standard to achieve a *q*-range of 0.045–1.5 Å^–1^. Two-dimensional images were recorded with exposure times of 600 s. The images were masked to remove the sample horizon, detector module gaps, and beamstop and radially integrated from the apparent beam center. Data correction and reduction were performed using the GIXSGUI MATLAB toolbox.^29^ Two-dimensional scattering data was reduced to one-dimensional via radial integration, which was performed with a mask to remove contributions from “hot pixels”, the substrate horizon, and reflected beam. Fitting was performed using the *SasView* software package,^28^ employing the face-centered-cubic paracrystal (colloidal crystal with optional packing disorder parameter, hereafter named FCC paracrystal) model.^30^ Full details on the FCC paracrystal scattering model employed to fit the experimental data are available in the Supporting Information.

## Results and Discussion

First, the relative aggregation versus dispersibility of the PbS-TET-CA QDs is discussed within a family of closely related polyacene derivatives (TIPS-Tc, TIPS-Ac and TIPS-FTA). The PbS-TET-CA QDs were obtained through ligand exchange of as-synthesized PbS-OA quantum dots. PbS-TET-CA QDs have previously been characterized via small-angle X-ray and neutron scattering, finding ligand shells packed with TET-CA that displaces some oleate and some solvent from the ligand shell via competitive adsorption, leaving behind some residual native oleate. Thus, the resultant PbS-TET-CA QDs have a surface character intermediate between oleate and TET-CA, which has proven to be an important factor in their self-assembly.^18^

**Figure 1:**
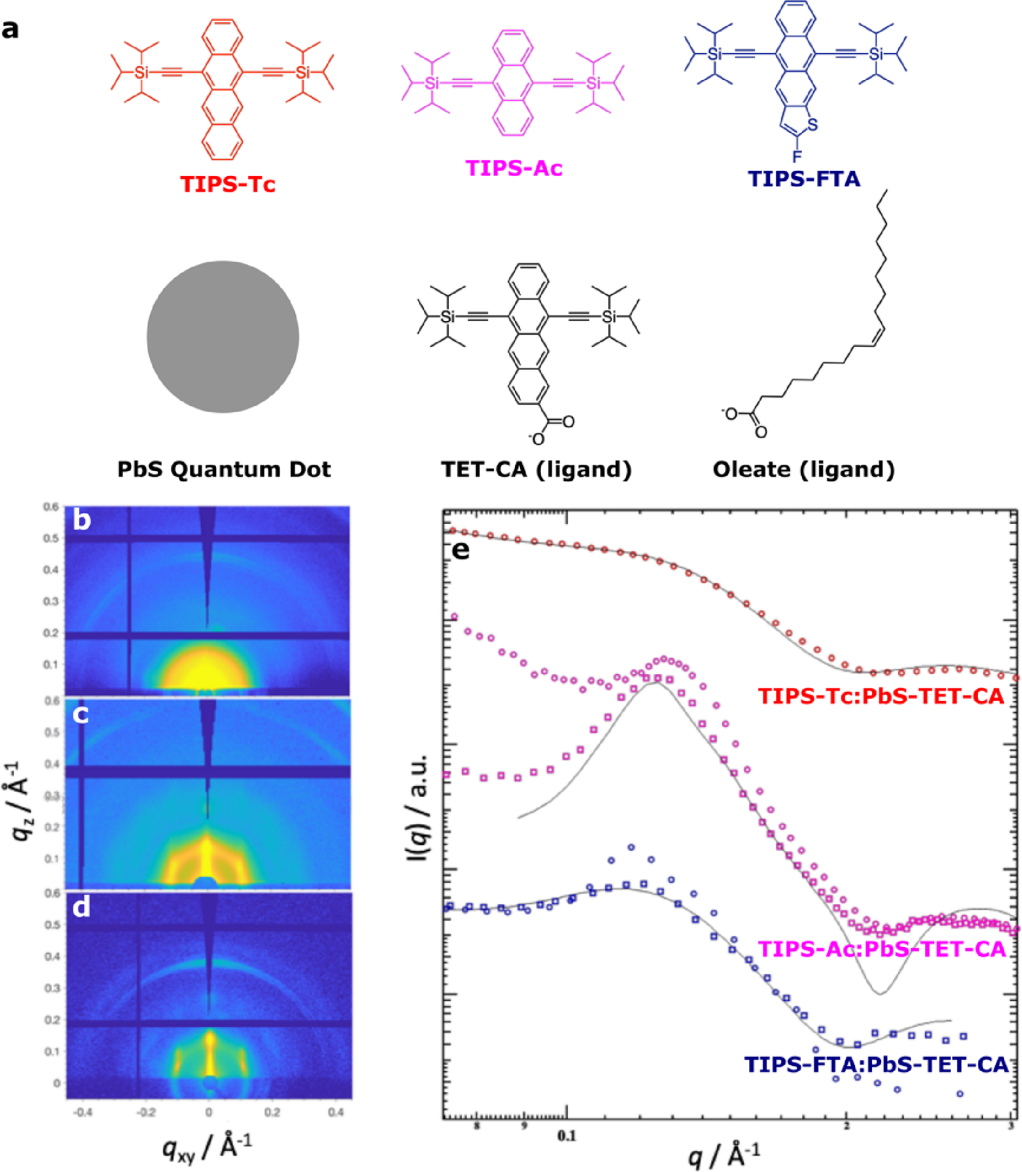
Structural studies on the effect of different small-molecule semiconductors TIPS-Tc, TIPS-Ac, and TIPS-FTA on QD ordering within hybrid small-molecule:QD thin films. (a) Schematic showing the materials employed: small-molecule host materials and TET-CA ligated PbS quantum dots. (b–d) 2D-GISAXS data for (b) TIPS-Tc:PbS-TET-CA, (c) TIPS-Ac:PbS-TET-CA, and (d) TIPS-FTA:PbS-TET-CA films. (e) Corresponding 1D radially integrated data for TIPS-Tc:PbS-TET-CA (red symbols), TIPS-Ac:PbS-TET-CA (magenta symbols), and TIPS-FTA:PbS-TET-CA films (blue symbols). The full radial integrations 0–180° are denoted by circles, and sectoral radial integrations between 45 and 90° are denoted by squares. The associated fits obtained using the FCC paracrystal model are described by solid black lines, with data scaled on the vertical axis for clarity.

The QD dispersibility within the TIPS-Tc, TIPS-Ac, and TIPS-FTA host matrices was characterized via GISAXS/GIWAXS with results presented in Figure 1b–e. Results show that for the TIPS-Tc:PbS-TET-CA film, QDs are relatively well-dispersed within the TIPS-Tc matrix as demonstrated by the absence of a structure factor/aggregation peak at ∼0.35 Å^–1^. The QD:TIPS-Ac and QD-TIPS-FTA films exhibit significantly worse QD dispersibilities as demonstrated by the clear structure factor/aggregation peak at ∼0.35 Å^–1^.

Further analysis of scattering data was performed to better quantify the QD dispersibilities for the QD:TIPS-Tc/TIPS-Ac/TIPS-FTA films. For the TIPS-Tc:PbS-TET-CA film, the 2D scattering pattern is highly isotropic, indicating homogeneous QD distributions throughout the bulk of the film. It should be noted that it was not possible to fit the 1D scattering data in the TIPS-Tc:PbS-TET-CA film to a sphere–hard sphere model, as would be expected for fully dispersed QDs (data for solution scattering of QDs in toluene is available in Figure S1 and corresponding fit parameters in Table S1). Fits capturing the most significant features of the 1D radially integrated scattering data were obtained employing a colloidal crystal model named as FCC paracrystal (with full fit parameters shown in Table S2 of the Supporting Information), where the QDs are described as being ordered on a face-centered cubic (FCC) lattice with paracrystalline distortion. The paracrystalline distortion factor is a convenient metric to quantify the QD ordering within the films. For a distortion factor of zero, all QDs are perfectly orientated on an FCC crystal lattice, with no deviation from their ideal positions, as would be the case for a defect-free QD superlattice. As the distortion factor approaches unity, the distribution of QDs becomes equivalent to that of the sphere–hard sphere scattering model with completely spatially disordered QDs possessing minimal long-range order correlations and no recognizable colloidal crystal structure. For further details regarding the scattering models employed in this work, please see the Supporting Information.

For both TIPS-Ac:PbS-TET-CA and TIPS-FTA:PbS-TET-CA films, the 2D GISAXS patterns (Figure 1c,d) show two distinct QD morphologies, with anisotropic scattering, including rod-type features (*q_xy_* ∼0.125 Å^–1^) and radial isotropic scattering rings (*q_r_* ∼0.125 Å^–1^). Fitting 1D radially integrated data (Figure 1e, circles) using previous models (sphere–hard sphere and FCC paracrystal)^18,31,32^ did not produce adequate results due to the anisotropic nature of the scattering patterns. Scattering rods arise from ordered correlations between scattering centers perpendicular to the substrate orientation,^33^ without in-plane correlations. For the TIPS-Ac:PbS-TET-CA and TIPS-FTA:PbS-TET-CA films studied here, such lateral correlations may be explained by the presence of an ordered monolayer of QDs at the substrate surface.

Simulations using BornAgain software were performed to provide additional qualitative evidence of the proposed contribution of the scattering rod features to the 2D GISAXS data. The simulations used a sphere*2D interference lattice model for the TIPS-Ac:PbS-TET-CA film, with both experimental data and simulations shown in Figure S2b,c.^34^

The experimental data does not exhibit scattering features as intense as those in the simulation, but the most pronounced rod features and their higher order reflections show sufficient similarity. These differences in intensity may be explained by the presence of disorder in the QD monolayer, which was not considered in the simulation. Therefore, the presence of rod-type scattering features qualitatively identifies one of the QD morphologies as a mostly well-ordered monolayer with some degree of disorder.

A second QD morphology was identified in the 2D GISAXS data for both TIPS-Ac:PbS-TET-CA and TIPS-FTA:PbS-TET-CA. To analyze this morphology, radial integrals were performed between 45 and 90° to exclude scattering from the ordered QD monolayer morphology’s rod-like scattering. The results of these integrals, shown in Figure 1e along with the corresponding fits using the FCC paracrystal model (represented by squares and solid lines, respectively), indicate that the second QD morphology consists of dispersed QDs within the OSC host matrix phase.

The relative dispersibility of PbS-TET-CA QDs follows the order TIPS-Tc (good QD dispersibility) > TIPS-FTA > TIPS-Ac (poor QD dispersibility), as shown by the lattice distortion factors of 0.32, 0.24, and 0.12 obtained from data fits. The TIPS-Ac:PbS-TET-CA and TIPS-FTA:PbS-TET-CA films have an ordered QD monolayer at the substrate–film interface, with the bulk QDs dispersed to varying extents within the OSC morphology.

The poor dispersibility of the PbS-TET-CA QDs observed within the TIPS-Ac and TIPS-FTA host matrices is disadvantageous for potential applications in photon multiplier film since a high proportion of QDs lie in close proximity leading to luminescence quenching.^19^ This is unlike the PbS-TET-CA:TIPS-Tc films, where significantly improved QD dispersibility is achieved within the crystalline TIPS-Tc matrix.

Irrespective of their intermolecular crystal packing motif, all OSCs investigated here self-assemble hierarchically into spherulitic crystalline morphologies spanning micrometer-length scales, as identified via polarized optical microscopy (Figure S3). It is well-established that small differences between similar chemical species often lead to different crystal packing motifs,^35−37^ as is the case for the three polyacene host materials investigated here. TIPS-Ac orients in a herringbone pattern, with either face-to-edge or slipped face-to-face stacking, depending upon the polymorph.^38^ The crystallization of TIPS-FTA is likely enhanced due to F–F and F–S interactions, as well as the known interactions between fluorinated and non-fluorinated aromatic surfaces,^39^ which tune the intermolecular order to favor π stacking in the solid state.^40^ Crystalline packing in TIPS-Tc is an intermediate between herringbone and brick-wall packing, with the molecules offset with little overlap of the tetracene backbones.^41^

The dispersibility of the QDs within the crystalline host OSC depends upon interactions between the QD ligands and OSC molecules. These interactions give the QDs potential to not only act as heterogeneous nucleating agents but also disrupt the large-scale molecular order of growing OSC crystals. Taking a Hansen solubility approach enables the relative miscibility of the QD ligands and OSC to be approximated via determination of the interaction radius, *R_a_*.^42,43^ QD ligands such as oleate typically have unfavorable interactions with the OSC, resulting in a large driving force for QD aggregation (as solvent evaporation proceeds and ligand–OSC interactions increase) that minimize interactions between the OSC and the QD ligands. On the other hand, quantum dot ligands that are chemically similar to the OSC (e.g., TET-CA) have reduced unfavorable interactions, leading to enhanced mixing of the OSC and QDs in solution.^18^ While the driving force for QD aggregation is reduced for TIPS-Tc/TIPS-Ac/TIPS-FTA due to the employment of a chemically similar polyacene carboxylic acid ligand, the OSC crystallization during solvent evaporation imparts additional complexity to this process. We speculate that here, the growing crystal is influenced not only by the specific intermolecular interactions between OSC molecules but also by the associated penalty for the growing OSC crystal front to either distort in the case of QD occlusion or drive expulsion of the QD “impurities”. The morphological insights into the TIPS-Ac:QD and TIPS-FTA:QD films indicate less well-dispersed QDs than in the TIPS-Tc:QD films, which most likely result from a greater propensity for the growing TIPS-Ac or TIPS-FTA crystals to expel quantum dots. This leads to poor quantum dot dispersibilities since in final film morphologies, the swept-out material tends to end up agglomerated at crystal boundaries and interstices.^44^

In the previous section, we discussed the reasons why alternative OSC hosts to TIPS-Tc tend to result in poor QD dispersibility. One possible solution to this problem could be to blend two different OSC hosts. The reasoning behind this choice is that TIPS-Ac has a strong tendency to crystallize, which we believe is a significant factor that affects QD dispersibility. Thus, by blending TIPS-Tc and TIPS-Ac together, we hope to suppress this crystallization tendency of TIPS-Ac and thus improve QD dispersibility within TIPS-Ac:PbS-TET-CA films.

Blends of TIPS-Tc:TIPS-Ac:PbS-TET-CA were spun-cast from toluene at 1500 rpm, with solutions containing a total OSC content of 100 mg mL^–1^ at TIPS-Ac weight fractions of 0.17, 0.33, 0.5, 0.66, and 0.83 and a PbS-TET-CA content of 10 mg mL^–1^. To reduce the length of abbreviations, we will adopt the following shorthand. TIPS-Tc:TIPS-Ac:PbS-TET-CA blend films will be referred to as Tc:Ac:QD films. A particular film in the series can be identified by its TIPS-Ac weight fraction, e.g., Ac_0.17_ for TIPS-Tc:TIPS-Ac:PbS-TET-CA with a TIPS-Ac weight fraction of 0.17 and so on.

**Figure 2:**
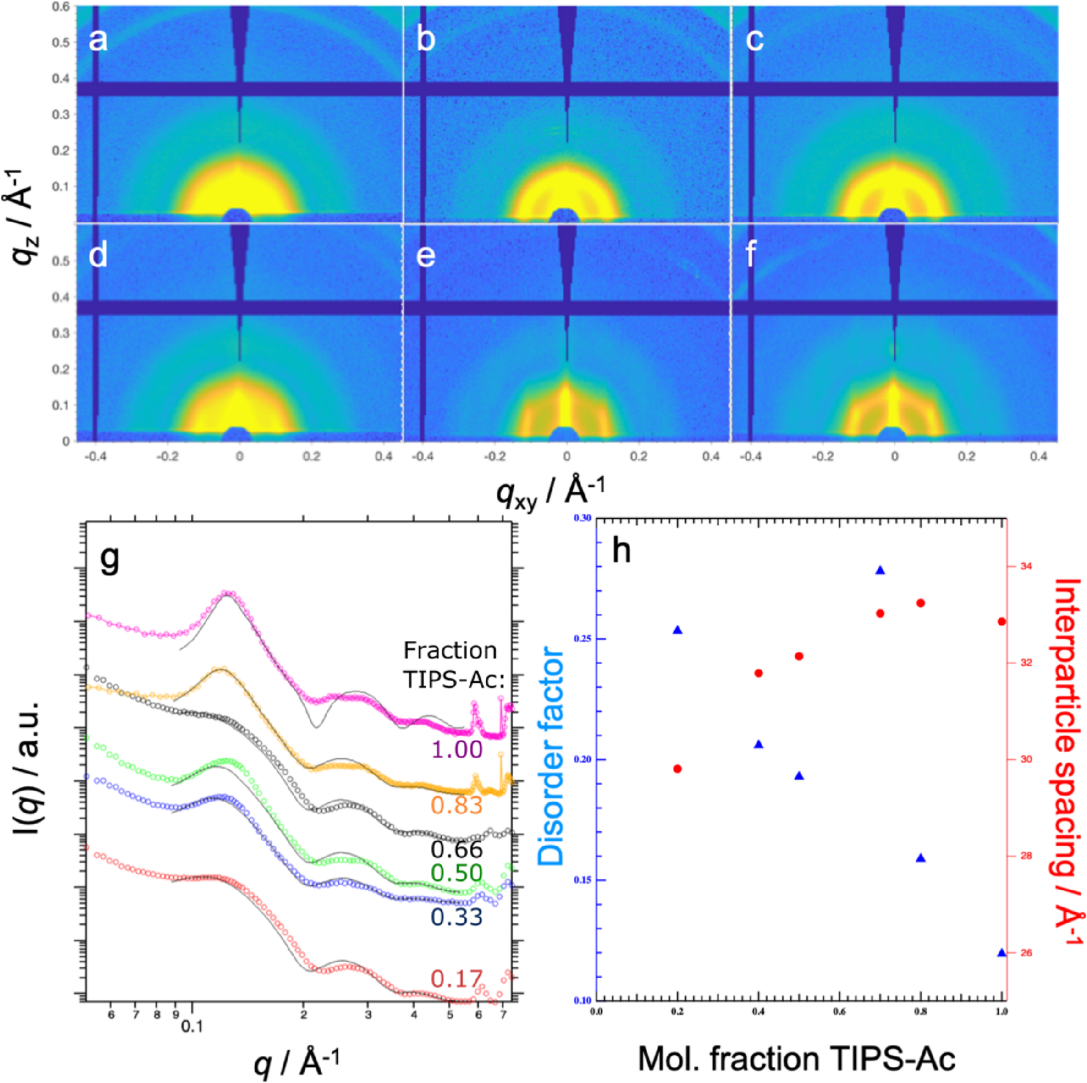
Structural studies of the effect of the TIPS-Tc:TIPS-Ac host phase on QD ordering within hybrid small-molecule:QD thin films. (a–f) 2D-GISAXS data for Tc:Ac:QD thin films Ac_0.17_, Ac_0.33_, Ac_0.5_, Ac_0.66_, Ac_0.83_, and Ac_1.0_ for (a–f), respectively. (g) Corresponding 1D radially integrated data for Ac_0.17_ (red circles), Ac_0.33_ (blue circles), Ac_0.5_ (green circles), Ac_0.66_ (black circles), Ac_0.83_* (orange circles), and Ac_1.0_* (magenta circles) and the associated fits (solid black lines) using the FCC paracrystal model, with data scaled on the vertical axis for clarity (*radial integral taken in the sector between 45 and 90° due to the presence of Bragg rod features). (h) Data derived from the fits in (g) showing the disorder factor and interparticle spacing as a function of TIPS-Ac weight fraction.

2D GISAXS data collected from the resulting films, along with 1D radially integrated data (for the Ac_0.83_, the radial integral was taken between azimuthal angles of 45 and 90° to remove contributions from rod-like scattering features), corresponding model fits (FCC paracrystal), and selected fit parameters, are shown in Figure 2 (with full fit parameters available in Table S3). At TIPS-Ac fractions <0.83, the 2D GISAXS data predominantly contain isotropic scattering features commensurate with a single QD morphology, while for the Ac_0.83_ blend film, 2D scattering data is largely reminiscent of that of the TIPS-Ac:PbS-TET-CA film, possessing both rod-like scattering features and isotropic scattering rings and is thus indicative of two distinct QD morphologies. First, QD morphologies of the Ac_0.17_, AC_0.33_, Ac_0.5_, and Ac_0.66_ are discussed. At the lowest TIPS-Ac content Ac_0.17_, scattering is well-described by the FCC paracrystal model with a high lattice disorder factor (0.25) and a lattice unit cell of 82.2 Å, which equates to an interparticle separation of 29.8 Å. As the weight fraction of TIPS-Ac is increased (Ac_0.33_ and Ac_0.50_), the QDs distributed with the FCC lattice are more ordered and consequently less well-dispersed within the OSC matrix phase as evidenced by the decrease in the magnitude of the disorder parameter. However, at Ac_0.66_, the scattering features are in fact more reminiscent of that of well-dispersed QDs with a disorder factor of 0.28, indicating a significant improvement in the dispersibility of QDs for this particular composition of the binary TIPS-Tc:TIPS-Ac matrix. As the TIPS-Ac weight fraction was increased further to Ac_0.83_ and Ac_1.0_, adequate fits to the FCC paracrystal model were only obtained through taking radial integrals at azimuthal angles between 45 and 90° (i.e., only within a particular sector to remove contributions from Bragg rods) and show further reductions in the lattice disorder parameter, indicating that further increasing the TIPS-Ac content reduces the QD dispersibility. While this approach allows comparison between the Tc:Ac:QD films, it should be noted that for Ac_0.83_ and Ac_1.0_, respectively, the scattering models employed do not account for the weak scattering peaks observed at *q =* ∼0.24 and 0.28 Å^–1^. For these blend films, scattering peaks at these *q* values in the blend films correspond to (022) and (113) peaks of an FCC colloidal crystal of QDs, suggesting the presence of another population of aggregated QDs. This implies that the disorder factor values are likely underestimated as the analysis is based on a single average QD morphology. Interestingly, as the TIPS-Ac content was increased from 0.17 to 1.0, the interparticle spacing increased from 30 to 33 Å. We ascribe this phenomenon to the slight difference in the interaction parameters between TIPS-Tc and TIPS-Ac, leading to a sharpening of the QD-ligand:OSC interface.

**Figure 3:**
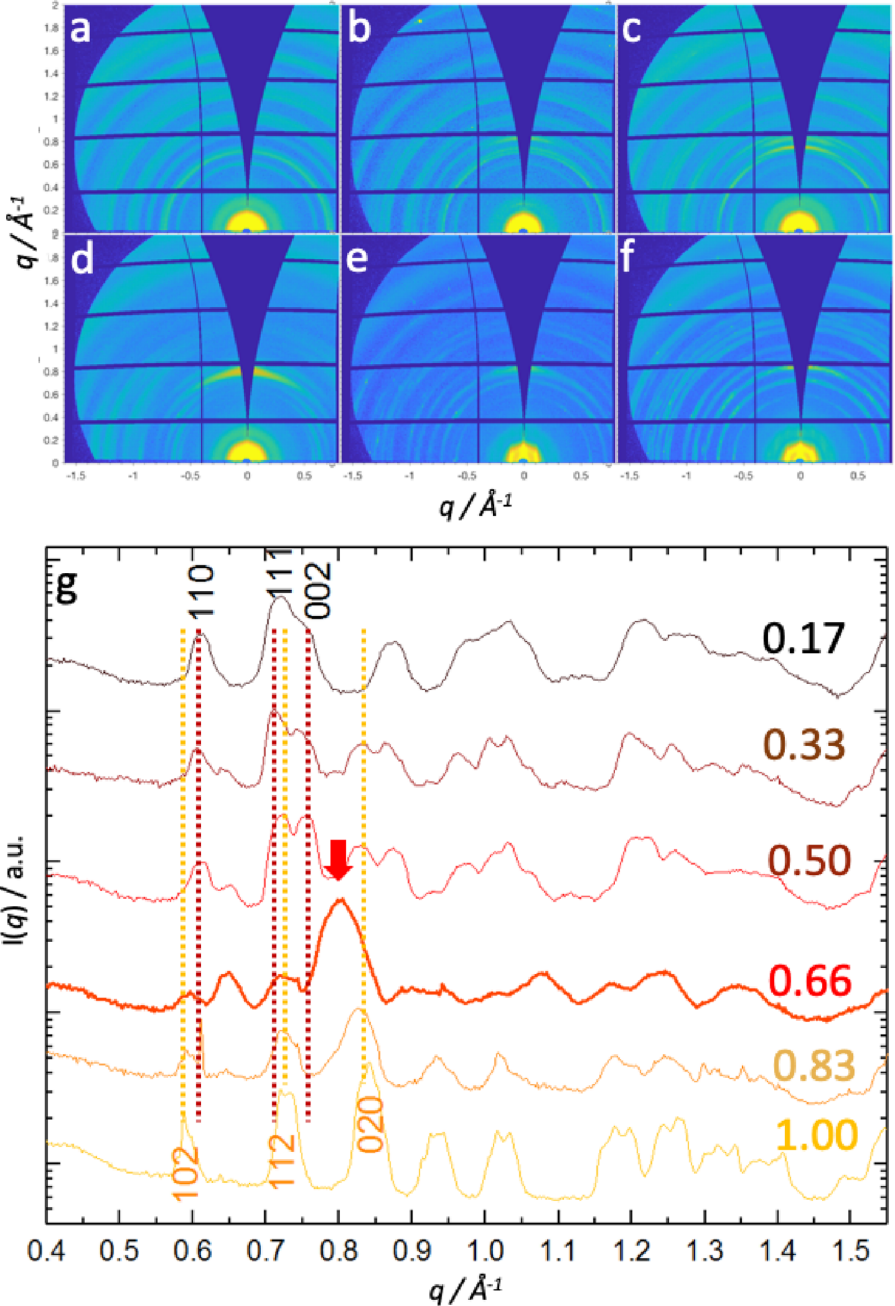
Structural studies of the morphologies of the TIPS-Tc:TIPS-Ac host phase for hybrid small-molecule:QD thin films. (a–f) 2D-GIWAXS data for Tc:Ac:QD thin films Ac_0.17_, Ac_0.33_, Ac_0.5_, Ac_0.66_, Ac_0.83_, and Ac_1.0_ for (a–f), respectively. (g) Corresponding 1D radially integrated data for Ac_0.17_, Ac_0.33_, Ac_0.5_, Ac_0.66_, Ac_0.83_, and Ac_1.0_ for (a–f), respectively, with the corresponding (110), (111), and (002) TIPS-Tc crystalline reflections and (102), (112), and (020) TIPS-Ac crystalline reflections showing contributions from the respective small-molecule components of the blend films.

GISAXS data for the Tc:Ac:QD composition series reveals interesting phase behavior between QD dispersibility and the weight fraction of TIPS-Ac. To gain a better understanding of this relationship, GIWAXS regions of the scattering patterns were analyzed. Specifically, we looked at GIWAXS regions of the obtained scattering patterns where *q_r_* > 0.6 Å^–1^, which are associated with the intermolecular packing of the OSC, with 2D scattering patterns shown in Figure 3a–f and the corresponding radially integrated scattering data shown in Figure 3g.

**Figure 4:**
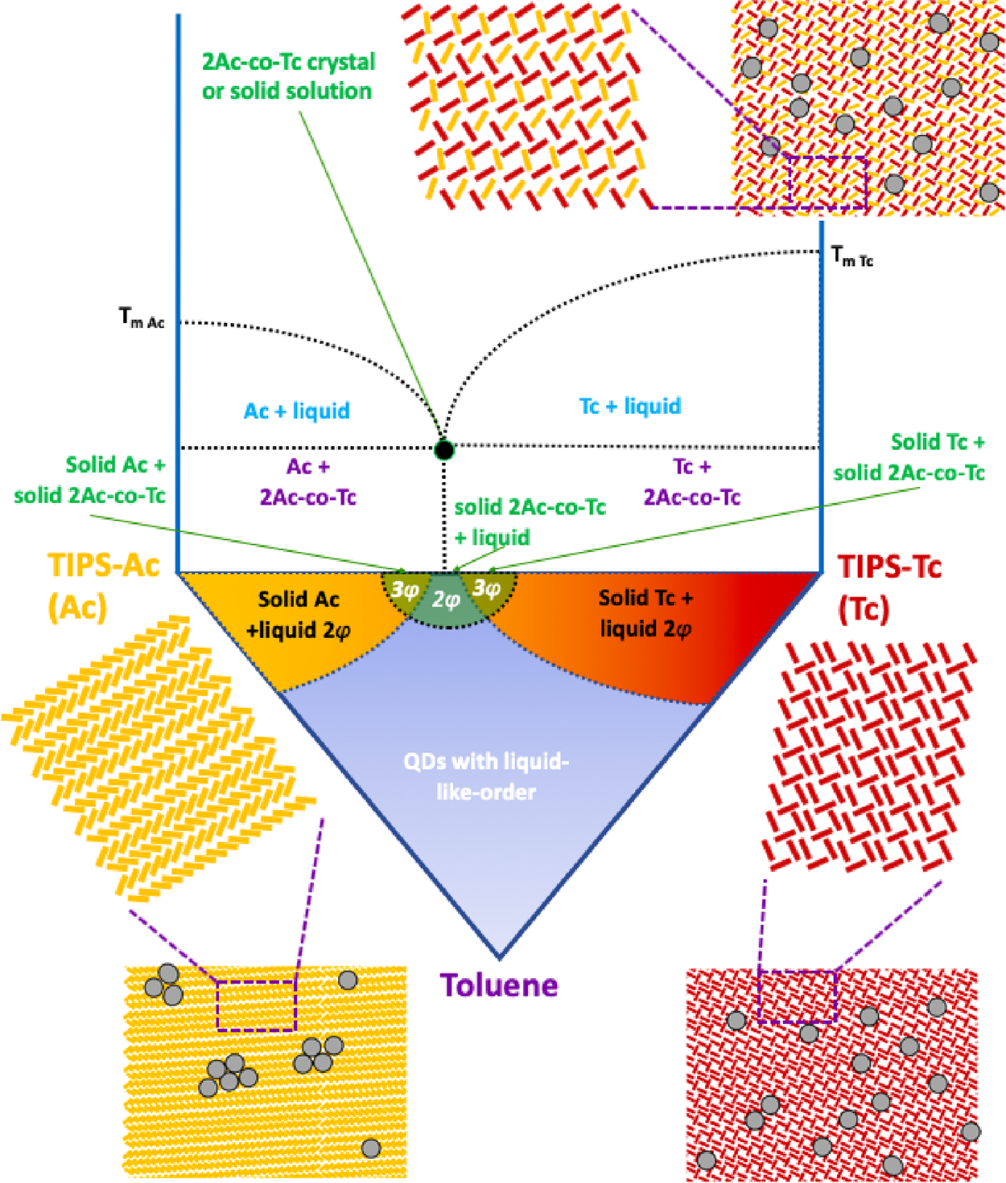
Inferred phase diagram for TIPS-Tc:TIPS-Ac:QD thin films. (Top) Solid temperature phase diagram. (Bottom) Ternary phase diagram, representing processing from the solution of Tc:Ac:QD thin films with illustrations of the pure TIPS-Ac and TIPS-Tc crystal phases and a third mixed TIPS-Ac-co-TIPS-Tc crystalline phase.

For the highest TIPS-Tc content film Ac_0.17_, the (110), (111), and (002) reflections are clearly identifiable (as listed for CSD deposition 962667 for pure TIPS-Tc). Meanwhile, for the highest TIPS-Ac content film Ac_1.0_, the (102), (112), and (020) reflections are also clearly identifiable (as listed for CSD deposition 962668). For all TIPS-Tc:TIPS-Ac compositions (apart from Ac_0.66_), the crystalline reflections may be ascribed to either TIPS-Tc or TIPS-Ac, with relatively distinct phases of each forming. However, for Ac_0.66_, new intense diffraction peaks at 0.65 and 0.82 Å^–1^ emerge that cannot be ascribed to either TIPS-Tc or TIPS-Ac crystalline morphologies. Closer inspection of data at 0.66 and 0.82 Å^–1^ for all the Tc:Ac:QD blends indicates the presence of a small fraction of such a co-crystalline TIPS-Ac-co-TIPS-Tc phase. The identified sample exhibiting the most pronounced TIPS-Ac-co-TIPS-Tc phase also exhibited the most effective QD dispersibility. This suggests that the co-crystal phase behavior suppresses the crystallization of the pure OSC components that would ordinarily lead to poor QD dispersibility. Thus, the observed blend morphologies may contain pure TIPS-Tc or pure TIPS-Ac with a TIPS-Ac-co-TIPS-Tc crystalline phase. From the observed scattering data for the TIPS-Tc:TIPS-Ac:QD films, we infer a potential phase diagram (Figure 4) for this system, illustrating the presence of pure TIPS-Ac and TIPS-Tc crystal phases and a third mixed TIPS-Ac-co-TIPS-Tc crystalline phase and how these impact upon QD dispersibility within the films.

## Conclusions

In this work, the dispersibility of QD functionalized with an organic semiconductor ligand within either TIPS-Tc, TIPS-Ac, or TIPS-FTA was investigated. Results show that the relative dispersibility of the QDs follows the order TIPS-Tc (good QD dispersibility) > TIPS-FTA > TIPS-Ac (poor QD dispersibility). We hypothesize that within OSC hosts possessing stronger interactions driving OSC crystallization, QDs become poorly dispersed as they are expelled from the growing OSC crystal, as is the case for TIPS-Ac and TIPS-FTA.

Aiming to overcome the poor QD dispersibility observed in TIPS-Ac:QD films, we explored the effect of blending OSC hosts (TIPS-Tc:TIPS-Ac). Our results show that within a narrow composition window on a sketched phase diagram, a TIPS-Ac-co-TIPS-Tc co-crystalline phase forms, within which good QD dispersibility is observed. These approaches outlined here show how co-crystal phase behavior may be a promising route for achieving optimal, dispersed QD morphologies for OSC:QD blend systems such as photon-multiplier materials and beyond.

## Supporting Information Available

Synthesis and further details on scattering and polarized optical microscopy (PDF)
