# Supplementary material for: Mixed Small-Molecule Matrices Improve Nanoparticle Dispersibility in Organic Semiconductor-Nanoparticle Films
Source: Langmuir. 2023 Mar 20;39(13):4799–808. doi: 10.1021/acs.langmuir.3c00152 (PMC10077578; doi:10.1021/acs.langmuir.3c00152)
Supplement: Supplementary file 1 — la3c00152_si_001.pdf [file la3c00152_si_001.pdf]

Supporting Information: Mixed small-molecule matrices improve nanoparticle dispersibility in organic semiconductor-nanoparticle films

Daniel T. W. Toolan*,[a] Michael P. Weir,[b,c] Rachel C. Kilbride,[a] John E. Anthony,[d] Neil C. Greenham,[e] Richard H. Friend,[e] Akshay Rao,[e] Oleksandr O. Mykhaylyk,[a] Richard A. L. Jones[f] and Anthony J. Ryan*.[a]

[a] *Department of Chemistry, The University of Sheffield, Dainton Building, Brook Hill, Sheffield, S3 7HF, UK*

[b] *Department of Physics and Astronomy, The University of Sheffield, Hicks Building, Hounsfield Road, Sheffield, S3 7RH, UK*

[c] *School of Physics and Astronomy, The University of Nottingham, University Park, Nottingham, NG7 2RD, UK*

[d] *University of Kentucky Center for Applied Energy Research, 2582 Research Park Drive, Lexington, Kentucky 40511, United States*

[e] *Cavendish Laboratory, Cambridge University, J. J. Thomson Avenue, Cambridge, CB3 0HE, UK*

[f] *John Owens Building, The University of Manchester, Oxford Road, Manchester M13 9PL.*

*Author to whom correspondence should be addressed.

DTWT: d.toolan@sheffield.ac.uk

AJR: a.ryan@sheffield.ac.uk

Synthesis

Solvents were purchased in bulk from VWR. Fluoro thiophene dialdehyde, anhydrous THF, and other reagents were purchased from Sigma Aldrich, except for 1,4-dihydroxynaphthalene which was purchased from TCI, and triisopropylsilyl acetylene, which was purchased from GFS chemicals. All chemicals were used as received. NMR spectra were measured on a Smart Probe-equipped 400 MHz Bruker Avance NEO instrument, and chemical shifts are reported in ppm and referenced to the deuterated solvents used.

2-fluoro thienoanthracene quinone:

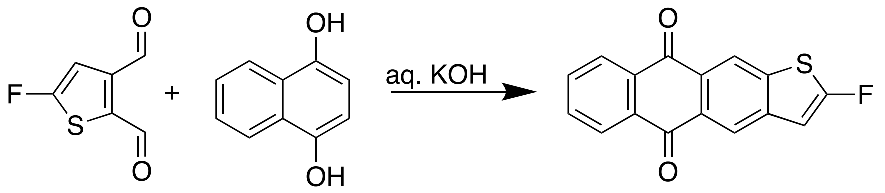

To a stirred solution of 0.5 g (3.16 mmol) 5-fluoro thiophene 2,3-dicarboxaldehyde and 0.56 g (3.5 mmol) of 1,4-dihydroxynaphthalene in ethanol (15 mL) was added five drops of 15% aqueous potassium hydroxide. After stirring for 30 minutes, the precipitate was filtered, and the solid cake washed first with methanol (200 mL) then with THF (100 mL) and finally with 50 mL of diethyl ether. The poorly soluble solid was allowed to dry in air overnight, and used without purification in the subsequent step.

TIPS-FTA

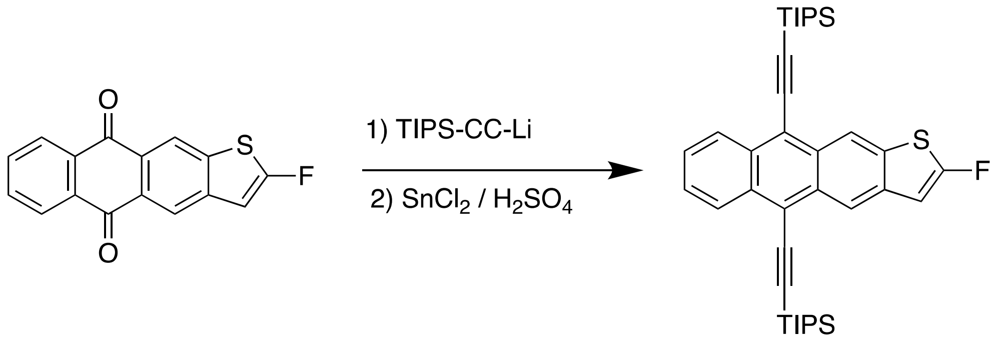

To a cooled (0 °C) solution of triisopropylsilyl acetylene (0.637 g, 3.5 mmol) in 10 mL of a 9:1 mixture of hexanes / anhydrous THF stirred in a flame-dried round-bottom flask was slowly added 3.2 mL of a 1.0 M solution of LiHMDS in THF. The solution was allowed to stir for 30 minutes, after which 0.282 g (1 mmol) of 2-fluoro thienoanthracene quinone was added in one portion. The reaction solution was stirred overnight, and then quenched with deionized water and extracted with hexanes. The organic layers were dried with magnesium sulfate and concentrated by rotary evaporation. The resulting crude diol was dissolved in a few mL of acetone and diluted with 10 mL of methanol open to air. To this, 6 equivalents of $SnCl_2$ dissolved in minimal 10% H_2SO_4 was added in one portion. The reaction solution was allowed to stir for 1 hour and then was extracted with hexanes. The organic layers were dried with magnesium sulfate and concentrated via rotary evaporation. The resulting solid was purified by chromatography on silica gel (hexanes) followed by recrystallization with acetone to give 0.45 g (80%) of the title compound. ^1H NMR (400 MHz, C_6D_6): δ 9.24 (s, 1H), 9.07 (s, 1H),

8.97-8.91 (m, 2H), 7.37-7-31 (m, 2H), 6.34 (d, J_{HH} = 2.56 Hz, 1H), 1.34-1.26 (m, 42H). ^{13}C NMR (101 MHz, $CDCl_3$): δ 167.51, 164.56, 137.16, 137.09, 134.45, 132.95, 132.68, 131.15, 130.66, 130.63, 128.70, 127.66, 127.55, 127.23, 127.17, 121.42, 121.34, 120.94, 118.89, 118.88, 118.27, 105.55, 105.50, 104.63, 104.42, 103.08, 102.97, 19.14, 19.10, 11.96, 11.92. ^{19}F NMR (376 MHz, C_6D_6): δ -117.68 HRMS (LDI): Calculated: 612.3078; Found: 612.3081.

Further details on scattering models

Sphere-hardsphere model (*sphere*hardsphere*)

In small-angle X-ray scattering (SAXS) of PbS nanocrystals suspended in an organic solvent such as toluene, the (organic) ligand shell and solvent are effectively contrast-matched and therefore scattering is dominated by the high electron density, metal-rich core. As PbS cores are quasi-spherical, they are adequately modelled as spherical scattering particles. The form factor of such particles, expressed as the scattering intensity as a function of the magnitude of the scattering vector, q, is given by

$$I(q) = \frac{scale}{V}\left[3V(\Delta\rho)\frac{\sin(qr) - qr\cos(qr)}{(qr)^3}\right]^2 + background$$

where r and V are the radius and volume of the sphere, $\Delta\rho$ is the scattering length density contrast difference between the solvent and the spherical particle.

The expressions for $I(q)$ above are for isolated scattering objects, and are known as form factors since they describe the scattering features that emerge from the object's intrinsic shape and form alone. However, in a many-particle system like a solution or nanocomposite of colloidal quantum dots, scattering may also arise from inter-particle correlations. This scattering is taken into account by a *structure factor* $S(q)$ that multiplies the form factor at each point in q, i.e.

$$I(q) = F(q)S(q)$$

For relatively low concentrations of PbS colloidal quantum dots in an organic solvent, say 1-10 mg mL^{-1}, the structure factor is a minor perturbation, such that it barely makes a visible adjustment to the data. If the system is sufficiently dilute it may be possible not to use the structure factor at all.

The hard sphere structure factor (as implemented in *SasView* version 4.2.2) is a calculation of the interparticle structure factor for monodisperse spherical particles interacting through excluded volume interactions. This is calculated using the Percus-Yevick closure[1] where the inter-particle potential is given as:

$$U(r) = \begin{cases} \infty & r < 2R \\ 0 & r \geq 2R \end{cases}$$

The sphere hard sphere scattering model was employed to fit the radially integrated scattering data for the batch of PbS quantum dots used in this work with data presented in Supplementary Figure 2 and corresponding fit parameters in Supplementary Table 1.

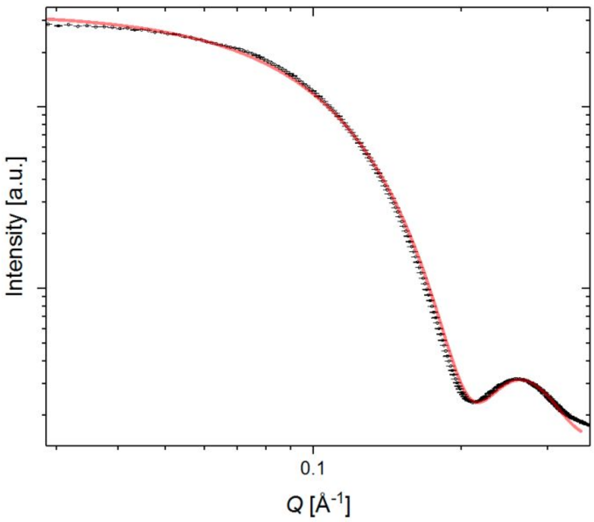

Figure S1. *Radially integrated solution scattering data for PbS-TET-CA dispersed in toluene (black circles), with the fit from sphere*hard sphere model (red line).*

Table S1. *Fit parameters from sphere-hardsphere model, data for PbS-TET-CA dispersed in toluene*

	scale	Back-ground	Sphere SLD (10^{-6} Å^{-2})	Solvent SLD (10^{-6} Å^{-2})	Sphere radius	radius.width	volfraction
PbS-TET-CA	972	126	50.7	10.2	20.7	0.1	0.0049

The PbS quantum dots employed in the TIPS-Tc:TIPS-Ac:PbS-TET-CA films were produced from a single batch, with fitting of solution scattering giving a value of r = 20.7 Å and a dispersity of 0.1 (expressed using the SasView convention as the standard deviation divided by the median). This dispersity is consistent with our previous work on PbS quantum dots and indicative of well-defined quantum dots possessing a narrow size distribution.[2-5]

Face-centred cubic lattice with paracrystalline distortion *model (FCC paracrystal)*

The *sphere-hardsphere* model is typically employed to describe the scattering from randomly ordered/dispersed quantum dots in solution. However, for the QD dispersibilities present in the TIPS-Tc, TIPS-Ac, TIPS-FTA and the TIPS-Tc:TIPS-Ac organic host matrices the *sphere-hardsphere* model insufficiently describes the scattering of the experimental data presented here.

The FCC paracrystal model (as implemented in *SasView* version 4.2.2) calculates the scattering from a face-centred cubic lattice with paracrystalline distortion. With the scattering intensity $I(q)$ calculated as:

$$I(q) = \frac{scale}{V_p} V_{lattice} F(q) Z(q) + background$$

where scale is the volume fraction of spheres, V_p is the volume of the primary particle, $V_{lattice}$ is a volume correction for the crystal structure, $F(q)$ is the form factor of the sphere (normalized), and $Z(q)$ is the paracrystalline structure factor for a face-centered cubic structure.

The lattice correction (the occupied volume of the lattice) for a face-centered cubic structure of particles of radius R and the nearest neighbour separation D is

$$V_{lattice} = \frac{16\pi}{3} \frac{R^3}{(D\sqrt{2})^3}$$

The distortion factor (one standard deviation) of the paracrystal is included in the calculation of $Z(q)$

$$\Delta a = \mathrm{gD}$$

where g is a fractional distortion based on the nearest neighbour distance. The distortion factor is a convenient metric to quantify the ordering of quantum dots in the films. For a distortion factor of zero all quantum dots are orientated on an FCC crystal lattice, with no deviation from their ideal positions and would be the case for a defect free quantum dot superlattice. As the distortion factor approaches unity, the distribution of quantum dots becomes equivalent to that of the *sphere-hardsphere* scattering model with completely dispersed / disordered quantum dots possessing minimal long range order correlations.

Table S2. Fit parameters for 1D radially integrated GISAXS data for TIPS-Tc:PbS-TET-CA, TIPS-Ac:PbS-TET-CA and TIPS-FTA:PbS-TET-CA films.

	scale	background	Lattice constant / Å	Lattice disorder parameter	Radius / Å	radius.width / Å	Sld / $\times10^{-6}$ Å^{-2}	sld_solvent / $\times10^{-6}$ Å^{-2}
TIPS-Tc:PbS-TET-CA	2.35	62.4	63.2	0.32	20.7	0.1	50.7	10.3
TIPS-Ac:PbS-TET-CA	10.00	10.00	85.22	0.12	20.7	0.1	50.7	8.30
TIPS-FTA:PbS-TET-CA	0.30	3.81	77.63	0.24	22.3	0.1	50.7	10.3

Table S3. Fit parameters for 1D radially integrated GISAXS data for TIPS-Tc:TIPS-Ac:PbS-TET-CA films.

Weight fraction TIPS-Ac	scale	background	Lattice constant / Å	Lattice disorder parameter	Radius / Å	radius.width / Å	Sld / $\times10^{-6}$ Å^{-2}	sld_solvent / $\times10^{-6}$ Å^{-2}

0.17	7.77	5.74	82.23	0.25	20.7	0.1	50.7	9.96
0.33	2.28	4.79	84.52	0.21	20.7	0.1	50.7	9.64
0.50	8.97	6.73	84.92	0.19	20.7	0.1	50.7	9.30
0.66	9.43	6.02	85.94	0.28	20.7	0.1	50.7	8.97
0.83	4.94	4.82	86.19	0.16	20.7	0.1	50.7	8.63
1.00	10.00	10.00	85.22	0.12	20.7	0.1	50.7	8.30

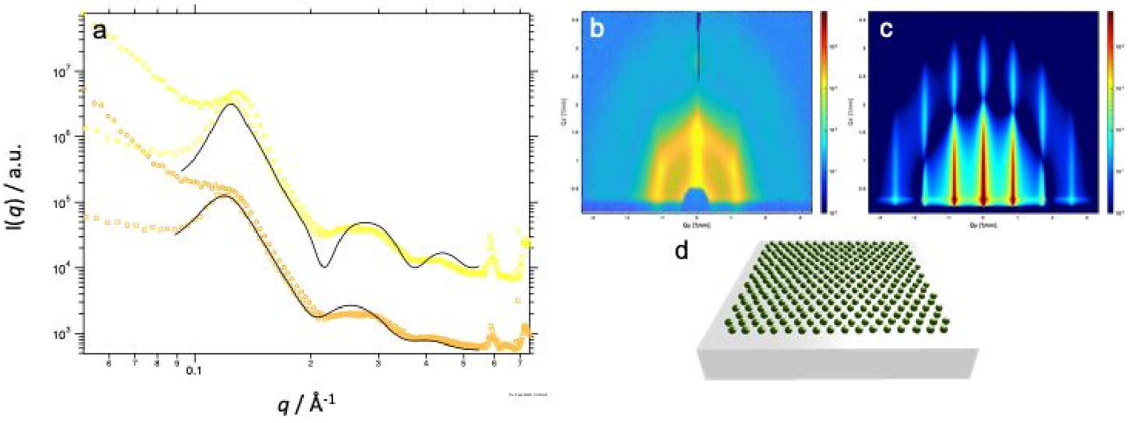

Figure S2. Structural studies on the effect of the TIPS-Tc:TIPS-AC host phase on QD ordering with hybrid small-molecule:QD thin-films. (a) 1d radially integrated data [at TIPS-Ac weight fractions of 0.83 (orange) & 1.0 (yellow), showing full radial integrations 0-180° (circles), partial radial integrations 45-90° (squares) and the associated fits (black lines) using an FCC colloidal crystal model, with data scaled for clarity. 2D GISAXS pattern (1.0 TIPS-Ac weight fraction b) with a simulation of an ordered monolayer QD colloidal crystal on the substrate surface, illustrating the origin of the "rod" type features observed at $q_{xy} = 0.12$ Å^{-1}.

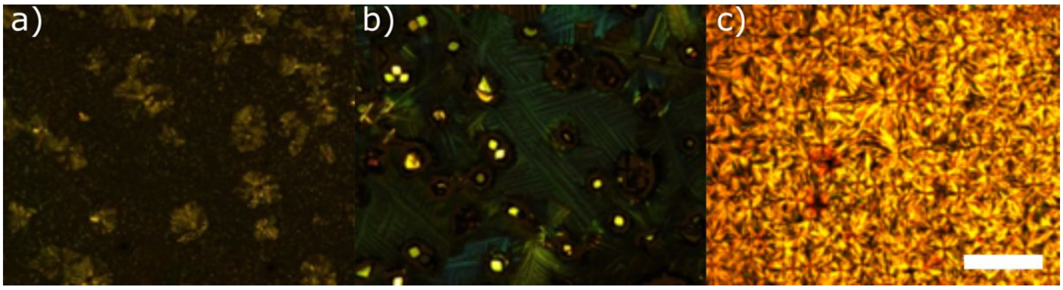

Figure S3. Polarized optical micrographs of (a) TIPS-Tc:PbS-TET-CA, (b) TIPS-Ac:PbS-TET-CA and (c) TIPS-FTA:PbS-TET-CA films, with a 500 μm scalebar.

References

(1) Percus, J. K.; Yevick, G. J. Analysis of Classical Statistical Mechanics by Means of Collective Coordinates. *The Physical Review* **1958**, *110* (1), 1.

(2) Toolan, D. T.; Weir, M. P.; Allardice, J.; Smith, J. A.; Dowland, S. A.; Winkel, J.; Xiao, J.; Zhang, Z.; Gray, V.; Washington, A. L. Insights into the Structure and Self-Assembly of Organic-Semiconductor/Quantum-Dot Blends. *Advanced Functional Materials* **2021**, 2109252.

(3) Toolan, D. T. W.; Weir, M. P.; Kilbride, R.; Willmott, J.; King, S. M.; Xiao, J.; Friend, R.; Rao, A.; Jones, R. A. L.; Ryan, A. J.; et al. Controlling the structures of organic semiconductor - quantum dot nanocomposites through ligand shell chemistry. *Soft Matter* **2020**, 10.1039/D0SM01109F. DOI: 10.1039/D0SM01109F.

(4) Allardice, J.; Gray, V.; Dowland, S.; Toolan, D. T. W.; Weir, M. P.; Xiao, J.; Zhang, Z.; Winkel, J. F.; Petty, A.; Anthony, J.; et al. Ligand Directed Self-Assembly of Bulk Organic-Semiconductor/Quantum-Dot Blend Films Enables Near Quantitative Harvesting of Triplet Excitons. *arXiv preprint arXiv:2009.05764* **2020**.

(5) Weir, M. P.; Toolan, D. T. W.; Kilbride, R. C.; Penfold, N. J. W.; Washington, A. L.; King, S. M.; Xiao, J.; Zhang, Z.; Gray, V.; Dowland, S.; et al. Ligand Shell Structure in Lead Sulfide–Oleic Acid Colloidal Quantum Dots Revealed by Small-Angle Scattering. *The Journal of Physical Chemistry Letters* **2019**, *10* (16), 4713-4719. DOI: 10.1021/acs.jpclett.9b01008.
